# Automated Transmission-Mode Scanning Electron Microscopy (tSEM) for Large Volume Analysis at Nanoscale Resolution

**DOI:** 10.1371/journal.pone.0059573

**Published:** 2013-03-26

**Authors:** Masaaki Kuwajima, John M. Mendenhall, Laurence F. Lindsey, Kristen M. Harris

**Affiliations:** 1 Center for Learning and Memory, The University of Texas at Austin, Austin, Texas, United States of America; 2 Section of Neurobiology, The University of Texas at Austin, Austin, Texas, United States of America; Virginia Tech Carilion Research Institute, United States of America

## Abstract

Transmission-mode scanning electron microscopy (tSEM) on a field emission SEM platform was developed for efficient and cost-effective imaging of circuit-scale volumes from brain at nanoscale resolution. Image area was maximized while optimizing the resolution and dynamic range necessary for discriminating key subcellular structures, such as small axonal, dendritic and glial processes, synapses, smooth endoplasmic reticulum, vesicles, microtubules, polyribosomes, and endosomes which are critical for neuronal function. Individual image fields from the tSEM system were up to 4,295 µm^2^ (65.54 µm per side) at 2 nm pixel size, contrasting with image fields from a modern transmission electron microscope (TEM) system, which were only 66.59 µm^2^ (8.160 µm per side) at the same pixel size. The tSEM produced outstanding images and had reduced distortion and drift relative to TEM. Automated stage and scan control in tSEM easily provided unattended serial section imaging and montaging. Lens and scan properties on both TEM and SEM platforms revealed no significant nonlinear distortions within a central field of ∼100 µm^2^ and produced near-perfect image registration across serial sections using the computational elastic alignment tool in Fiji/TrakEM2 software, and reliable geometric measurements from RECONSTRUCT™ or Fiji/TrakEM2 software. Axial resolution limits the analysis of small structures contained within a section (∼45 nm). Since this new tSEM is non-destructive, objects within a section can be explored at finer axial resolution in TEM tomography with current methods. Future development of tSEM tomography promises thinner axial resolution producing nearly isotropic voxels and should provide within-section analyses of structures without changing platforms. Brain was the test system given our interest in synaptic connectivity and plasticity; however, the new tSEM system is readily applicable to other biological systems.

## Introduction

Serial thin sections of <100 nm thickness have been used to visualize and reconstruct cellular and subcellular structures in the three-dimensional (3D) context from a wide variety of biological systems. Examples include, but are not limited to, Gram-negative bacteria [1], yeast [2], algae [3], nematode [4], lobster [5], frog [6], mouse [7], rat [8]–[10], and human cells [11]. In the central nervous system, serial section (ss) EM provides sufficient resolution to reveal cellular and subcellular structures within the three dimensional context of the surrounding neuropil, including dendrites, axons, and astroglial processes. In the last decade, ssEM has become widely recognized as a crucial tool to map and understand synaptic circuitry in the brain [12]–[16].

Our laboratory and others have used ssEM to understand how the structure of synapses and neuropil is modified by experience and in models of learning and memory [17]–[19] or under pathological conditions [20]–[26]. The results from these studies have provided fundamental insights into the anatomical substrates for changes in information processing and behavioral output. Both normal and pathological changes in neuronal morphology can involve subcellular structures such as, polyribosomes, microtubules, endosomes, dense core vesicles, and smooth endoplasmic reticulum, that require ssEM at nanoscale lateral resolution (<2 nm per pixel in x–y) to be reliably identifiable. Although microtubules and other small organelles have been detected at lower image resolutions using other ssEM techniques (e.g., [27], [28]), our experience is that reliable identification and quantification becomes difficult at lower resolutions [17], [19], [29], [30].

The ssEM approach with biological tissue has been implemented using transmission EM (TEM) on heavily en bloc and post-section stained specimens, because TEM affords the high lateral resolution required for the analysis of nanoscale subcellular structures. The tissue volume is generated by stacking and aligning the images of single or montaged fields across hundreds of serial ultrathin (∼45–70 nm) sections of plastic-embedded tissue on electron-transparent support films spanning slot grids [31]. However, TEM imaging suffers from a relatively small individual field size (∼100 µm^2^, or 10 µm per side), and therefore requires montaging with substantial data redundancy and electron dosage to achieve large image volumes. Manual exchange of specimen grids also adds to the cumbersome nature of TEM imaging for large scale analyses even with automated montaging [15], [32], [33].

Renewed interest in ssEM as a high-resolution 3D tool for neuroscience has led to improvements over the last decade in this otherwise time-, skill-, and labor-intensive approach [34], [35]. Recent studies [16], [18] have benefitted from newly developed methods based on an SEM platform using backscatter imaging from a tissue block surface that is successively removed by the diamond knife (serial block-face SEM, or SBFSEM; [36]) or a focused ion beam (FIB-SEM; [37], [38]). Unfortunately, these approaches may not yield the level of lateral resolution or contrast necessary for unequivocal identification of the nanoscale subcellular structures as discussed above. Furthermore, these approaches are destructive, so that sections cannot be retrieved for subsequent viewing at higher resolution.

We sought to improve ssEM by increasing the size of single image fields, while maintaining the needed lateral resolution and image quality, and reducing operator time and instrument cost. These goals were achieved by imaging serial ultrathin sections with a transmitted electron detector mounted on a field-emission (FE) SEM [39], that we call “transmission-mode SEM” or “tSEM” to differentiate from scanning transmission EM based on a TEM platform commonly referred to as “STEM” [40]. Transmission imaging in a SEM platform (sometimes referred to as “STEM-in-SEM”) has long been in use [41], but image resolution was unsatisfactory when compared to TEM and large frame storage at high resolution was not possible. Recently, field emission sources have become more commonly available for SEM instruments, contributing to an increase in beam brightness and image resolution [41]. The use of “STEM-in-SEM” imaging was previously proposed for polymer characterization as a more practical and affordable alternative to TEM imaging (e.g., [42]), although this prior approach lacked the large frame image acquisition. Our tSEM system has robust automated control of the specimen stage translation and electron beam scan dimension and rotation over large areas. The tSEM method is capable of acquiring single-frame images that can be more than 60 times larger than those taken with a TEM, while easily achieving the required 2 nm pixel size. In addition, tSEM imaging induces far less specimen drift and charging than the TEM, resulting in less physical distortion of the sections. Furthermore, the cost of this new system is substantially less than that of modern TEM or SEM-based ssEM systems (i.e., SBFSEM and FIB-SEM). We compare image quality between the new tSEM system and our modern TEM to analyze synapses and subcellular components as a basis for understanding synaptic connectivity and plasticity in the complex neuropil of the brain. The results show outstanding images that can be readily aligned using a new automated elastic alignment tool in TrakEM2 [43].

## Materials and Methods

### Tissue Sample Preparation

All animal procedures were performed in accordance with the Guide for the Care and Use of Laboratory Animals of the National Institutes of Health. The animal protocols were approved by Institutional Animal Care and Use Committee of The University of Texas at Austin (protocol numbers: 06062801 and AUP-2010-00181) and the Otago University Animal Ethics Committee (protocol number: 115/09). Hippocampal dentate gyrus tissue was obtained from adult rats that were rapidly perfusion-fixed with 2% formaldehyde and 2.5% glutaraldehyde (both aldehydes from Ladd Research, Williston, VT) in 0.1 M cacodylate buffer (pH = 7.35–7.4) under halothane anesthesia and tracheal supply of oxygen. Hippocampal area CA1 tissue was obtained from an acute brain slice (350 µm thickness) prepared from a rat under isoflurane anesthesia. The acute slice was recovered in oxygenated artificial cerebrospinal fluid for 3 hr before being fixed with 2.5% formaldehyde and 6% glutaraldehyde in 0.1 M cacodylate buffer (pH = 7.4) in a microwave oven [44]. The fixed tissue was then cut into slices (70 µm thickness) with a vibrating blade microtome (Leica Microsystems, Buffalo Grove, IL) and processed for electron microscopy as described previously [31], [45]. Briefly, the tissue was treated with reduced osmium (1% osmium tetroxide and 1.5% potassium ferrocyanide in 0.1 M cacodylate buffer), followed by microwave-assisted incubation in 1% osmium tetroxide under vacuum. Then the tissue underwent microwave-assisted dehydration and *en bloc* staining with uranyl acetate in ascending concentrations of ethanol. The tissue was embedded into LX-112 epoxy resin (Ladd Research) at 60°C for 48 hr before being cut into series of ultrathin sections at the nominal thickness of 45 nm with a 35° diamond knife (DiATOME, Biel, Switzerland) on an ultramicrotome (Leica Microsystems). The thickness of 45 nm was chosen to minimize overlap among small organelles within individual ultrathin sections (e.g. small synaptic vesicles are ∼30 nm in diameter). The serial ultrathin sections were collected onto Synaptek® Be-Cu slot grids (Electron Microscopy Sciences, Hatfield, PA, or Ted Pella, Redding, CA) coated with Pioloform F (Ted Pella), and stained with a saturated aqueous solution of uranyl acetate followed by lead citrate [46]. The SynapTek grids are thicker and more durable than TEM grids made only of Cu, and thus much less prone to damage even after handling multiple times. These specimens imaged at 28 kV, a relatively high accelerating voltage for SEM, displayed no charging and hence required no carbon coating or low vacuum operation. A typical series for our experiments consists of at least 200 serial sections collected on about 12 grids.

### Serial Section EM Imaging

The serial ultrathin sections were imaged with either a JEOL JEM-1400 TEM (Tokyo, Japan) or a Zeiss SUPRA® 40 field-emission (FE) SEM (Oberkochen, Germany). The TEM is equipped with a charge coupled device (CCD) camera with the field size of 4,080×4,080 (or 16.65×10^6^) pixels (Gatan UltraScan 4000; Pleasanton, CA), controlled by DigitalMicrograph software (Gatan). For TEM, the slot grids containing serial ultrathin sections were loaded into grid cassettes that were individually loaded into a Gatan 650 CC specimen holder that allow the grid to be rotated inside the chamber. The holder accommodates one grid at a time, and requires manual exchange between grids. At 6,000×magnification at 2 nm pixel size with the accelerating voltage of 120 kV, serial section images were manually acquired as the Gatan proprietary.dm3 files, which were later batch converted into 8-bit JPEG files with DigitalMicrograph software. Conversion into JPEG was originally done to save space in our database. No practical differences in identification of key structures were found compared to the same.dm3 images converted into TIFF format.

**Figure 1:**
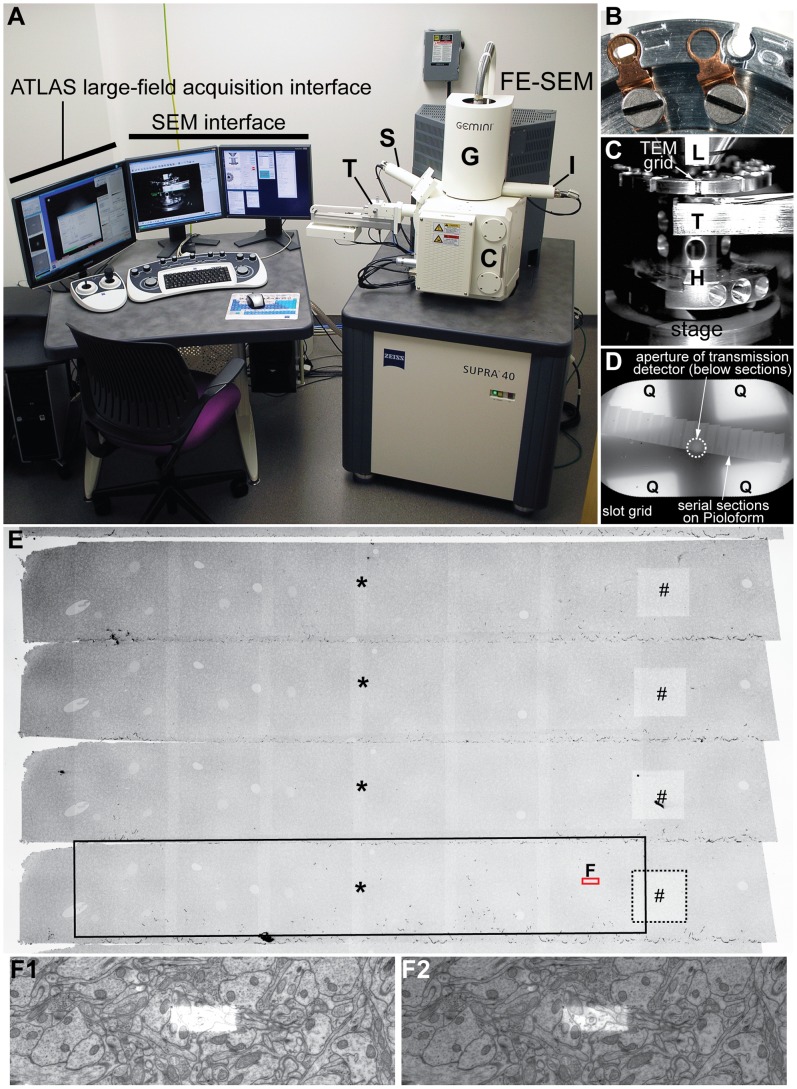
tSEM instrumentation. A: Zeiss SUPRA 40 field emission scanning electron microscope equipped with a secondary electron detector (S), an in-lens detector (I), and a retractable detector for transmitted electrons (tSEM detector; T). The column (G) contains the gun assembly and objective lenses. The specimen chamber door (C) slides open outward with the stage. The SEM is controlled through the SEM interface and console of keyboard, mouse, joysticks, or the integrated ATLAS system for large-field imaging. The SEM can also be fitted with a backscatter electron detector (not shown). B: Top view of the specimen holder magnified to show two of the grid holding positions (10 and 11). Position 10 is empty and the copper clip is disengaged to the left of the slot, while position 11 contains a TEM grid (3 mm diameter) with the clip engaged. C: TV camera view of the specimen chamber showing the arrangement of the final lens (L), tSEM detector (T), and sample holder (H) on the stage. Working distance is 4–5 mm between the final lens and the specimen, and 4–5 mm between the specimen and the detector. Chamber vacuum is maintained at <10^−7^ Pa during imaging. D: Low-magnification tSEM image of an entire slot grid containing serial sections. Below the sections, the aperture of the tSEM detector can be seen (circle with dotted line), which must to be aligned to the center of the image field. Four quadrants (Q) of the detector element are also used for imaging by collecting electrons scattered at higher angles. Imaging mode (normal or inverted) can be set for each detector element on the SEM interface. E: SEM secondary electron image of another set of serial sections (different from that shown in D). Each section measures about 510 µm width×71 µm height. This image was taken after acquisition of two image series, one consisting of single frame images (32 µm×32 µm surrounding the #) and the other consisting of mosaic images (6 columns×1 row; 360 µm width×64 µm height, surrounding the *). These image fields are seen as brightened areas on each section (outlined by dotted and black boxes in the bottom section). Regardless of the target image size (single or mosaic field), the operator is required to mark only the center of each field (indicated by “#” or “*”,) to set up the serial image acquisition. The area outlined by a red box (“F”) is further magnified in F1–2. F1–2: Magnified view of a subfield measuring 10.8 µm×3.6 µm around the center of the image tile indicated in E. The brightened area in the center is where repeated scans took place during the autofocus routine. If the image brightness is adjusted to the entire field, the autofocus area becomes too bright to discern ultrastructure within this area (F1). As demonstrated in F2, however, these repeated scans during the autofocus routine do not cause loss of the underlying tissue structure. The original tSEM image was acquired as a 16-bit TIFF file at 2 nm pixel size. The image brightness was optimized for either the entire image field or the autofocus area to generate the images in F1 and F2. These images were then converted to 8-bit TIFF files and down-sampled to 12 nm pixel size for the final figure.

The FE-SEM is equipped with a retractable multi-mode transmitted electron detector (“T” in Fig. 1A) and the integrated module called ATLAS™ (AuTomated Large Area Scanning; software version 3.5.2.385), which is a package of hardware and software designed to control scan generation, stage translation, and serial acquisition of large-field images (Fig. 1A). Multiple TEM grids containing serial sections were loaded into a single specimen holder (Fig. 1B, C). The “Zeiss Multi-Mode” transmitted electron detector is composed of the center aperture and four quadrants (Fig. 1D); for tSEM imaging, the imaging mode was set to ‘normal’ (for bright field detection behind the central aperture) and ‘inverted’ (for dark field), respectively. For serial tSEM imaging, the center of the region of interest was marked manually on each section throughout the entire series using the ATLAS system (Fig. 1E); for 200 serial sections this process took less than a half day. Then the ATLAS system automatically translated the stage, rotated the scan field if necessary, and acquired single-frame serial images (section-to-section and grid-to-grid) of up to 32,768×32,768 (or 1.07×10^9^) pixels with the transmitted electron detector at the pixel size of 2 nm (i.e., scan area = 4,295 µm^2^). The scan beam with a high brightness generated by the field-emission source is a critical component in achieving the level of lateral resolution demonstrated by this tSEM system. Pixel size can be adjusted depending on the operator’s needs; however, the limits on image resolution (i.e., the smallest distance between two points that can be visualized) was about 1.5 nm based on our experience with this system. The scan beam was set for a dwell time of ∼1.3 µs, with the accelerating voltage of 28 kV in high-current mode. Focus and brightness were also adjusted automatically with ATLAS. The acquired serial images were saved as 8-bit TIFF files, although as noted in the Results, 16-bit TIFF files retained image quality in the brightened area where focus was obtained from multiple scans (Fig. 1F).

If the size of each image field needs to be extended beyond 32,768×32,768 pixels, the operator can set up mosaics by specifying the target dimensions of the image field and the amount of overlap between image tiles. ATLAS then automatically determines the number of image tiles per field, based on the pixel size and the size of each image tile. For example, an image field of 360 µm wide×60 µm tall can be set up as a 6×1 mosaic of image tiles measuring 32,768×32,768 pixels each at 2 nm pixel size (Fig. 1E). The operator is required to mark only the center of mosaic field (* in Fig. 1E), versus (# in Fig. 1E) in the single-frame images. The ATLAS system can also be used to acquire large frame images with secondary detectors (on our system; Fig. 1A) or backscatter detectors (not on our system). On occasions where we needed lower magnification views of the overall grid or section (e.g., Fig. 1E), the specimens were imaged with secondary electron detectors mounted on the side of the chamber or inside the final lens (see Fig. 1A).

Serial EM images were aligned automatically using Fiji with the TrakEM2 plugin (http://fiji.sc, http://www.ini.uzh.ch/~acardona/trakem2.html) [43], [47], [48]. The images were aligned rigidly first, followed by application of affine and then elastic alignment. TrakEM2 was also used to generate single images from mosaics of image tiles. The aligned series was then imported into RECONSTRUCT™ software (http://synapses.clm.utexas.edu/) [49] to compare images acquired by the two EM platforms.

For preparation of figure plates, brightness and contrast of EM images were adjusted with either Fiji or Adobe Photoshop CS4 Extended (San Jose, CA). The original image pixels were retained unless otherwise noted in figure captions. Images from distortion analysis (see below) were generated in Matlab (version R2011b; MathWorks, Natick, MA). Final figure plates were prepared with Adobe Illustrator CS4.

### Image Distortion Analysis

**Figure 2:**
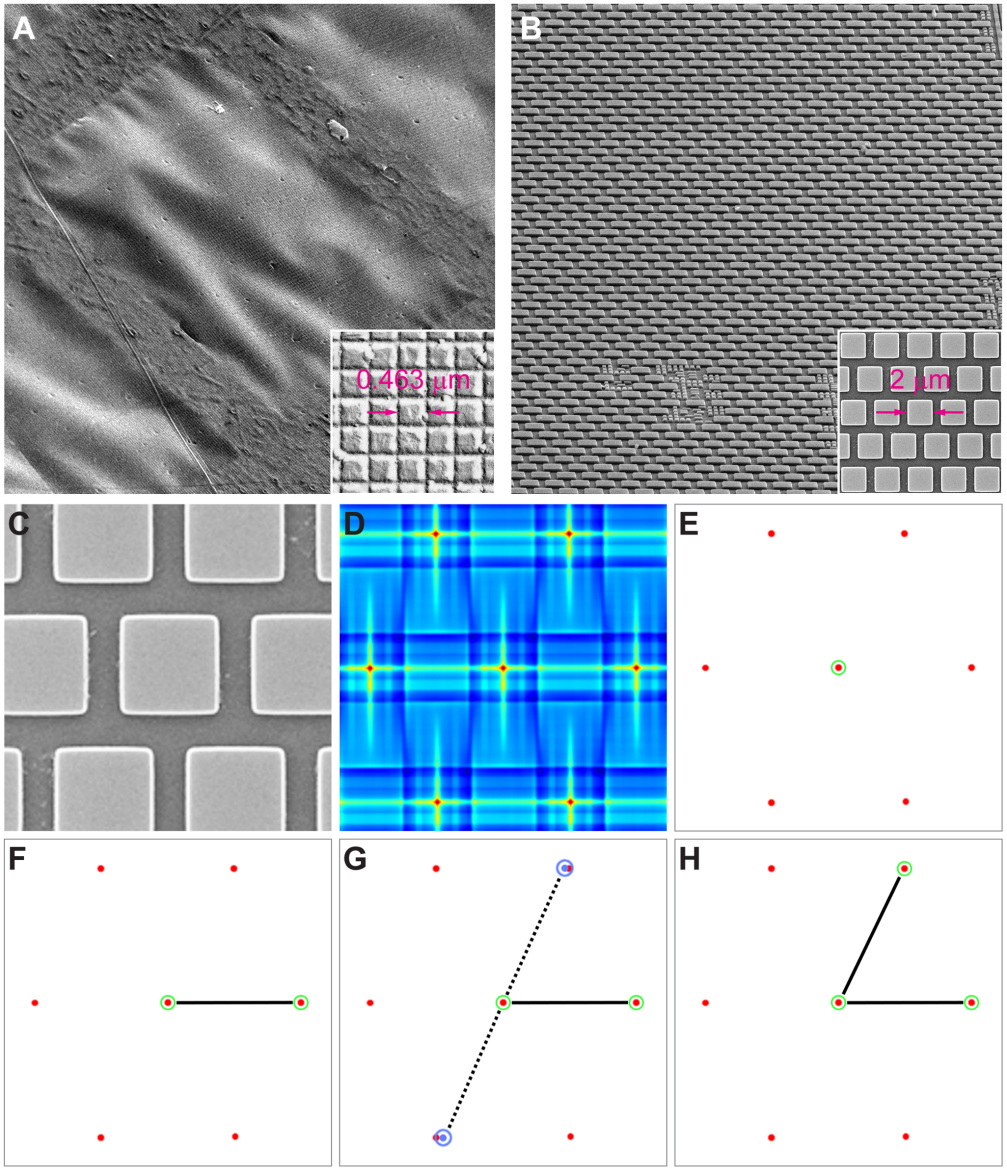
Distortion analysis method. A: SEM secondary electron image of TEM calibration standard (crossed diffraction grating replica) on a 300-mesh grid. The grid is tilted at 20° shows a wrinkled surface (tilt axis = upper left to lower right, with some scan tilt correction applied). Image field is about 56 µm per side, as in figure A. Inset: Details of the grating replica. Note that each square measures 0.463 µm×0.463 µm. This tSEM image was taken without any tilt. B: SEM secondary electron image of an integrated circuit (IC) chip used for evaluation of SEM scan distortion. The chip is tilted at 65° (tilt axis = left to right, dynamically focused, no scan tilt correction applied) to illustrate flatness. Image field is about 56 µm per side, which is approximately the same size as tSEM images. Inset: Details of the IC chip. Note that each square-shaped element measures 2 µm×2 µm, and is arranged in hexagonal arrays. This SEM secondary electron image was taken without any tilt. C: SEM secondary electron image of IC chip as in A, cropped to correspond with illustrations in D–H. D: Energy map created from the normalized cross-correlation of an image of an individual IC unit with the original image. E: The energy map is thresholded and peaks selected from within the resulting connected components. For each peak, as shown encircled in green, we build a triplet model to use for rejecting false detections and for extracting the true regular pattern. F: The detected location is paired with its nearest neighbor to form a line segment. G: We form putative neighbor locations at expected angles above and below the line segment (+60° and −120°, respectively). H: Here, the distance to the detection closest to the upper putative neighbor was smaller than for the lower one. The upper neighbor is selected to complete the triplet.

We had hoped to use a carbon replica grating to compare high-order distortions in the TEM and tSEM; however, the tSEM scan field is much larger than the individual grid support window, within which the grating replica also revealed large scale wrinkles (Fig. 2A). Instead, we imaged an unused integrated circuit (IC) chip, which has a regular pattern and is etched onto a very stiff substrate and is therefore very flat. The substrate is electron opaque, which makes the IC ineligible for use in calibrating the TEM. In the tSEM, we may image it under the same conditions that we might use for transmission imaging, with the exception that we use a secondary electron detector (Fig. 2B). We wrote software in Matlab to measure the high-order geometric distortion due to imaging with an electron microscope (available from: https://github.com/larrylindsey/MatlabCode/tags). Unfortunately, we have no prior knowledge to guide our expectation of how the sample should appear. Visual inspection tells us that the pattern consists of squares that are regular over a parallelogram with an inner angle of approximately 60° (Fig. 2C). To estimate the imaging distortion, we found the imaged locations of the units of this pattern and compared them to their expected locations. A match kernel representing a single unit was selected manually from the image and used for normalized cross-correlation with the original image, resulting in a map in which local maxima represent the precise image locations of these repeated units (Fig. 2D). To extract the maxima, we perform a simple threshold (Fig. 2E). Thresholding is advantageous in that it is simple to implement, but may occasionally result in spurious detections. We correct for these and extract our model for the expected pattern at the same time using RANSAC (RANdom SAmple Consensus) [50].

For each detected location **x_i_** (Fig. 2E), we find the nearest neighbor, **x_i,r_** (Fig. 2F). We form a triplet by finding the point **x_i,l_** at as close to the expected angle and distance from **x_i,r_** about **x_i_** as possible. In the case of a rectangular pattern, this angle would be 90°, but in our case this is +60° or −120° (Fig. 2G). Without loss of generality, we may orient the triplet **[x_i,l_, x_i_, x_i,r_]** such that **x_i,l_** is offset in approximately the same direction (positively or negatively) from **x_i_** for each *i*, and similarly for **x_i,r_** (Fig. 2H). Now, the average sign corrected vectors **g_i,r_** = **x_i,r_** − **x_i_** and **g_i,l_** = **x_i,l_** − **x_i_** yield a potential model for the regular pattern that we refine using RANSAC.

For a given population, the RANSAC algorithm attempts to find the largest subpopulation that fits a given model as follows: first, a random sample of the original population is taken and a model is fit to it. Each remaining member of the original population is tentatively added to this consensus sample. If it fits the model to a given certainty, it is kept. The model that yields the largest consensus sample is said to be the correct one, and is said to fit its corresponding sample population. In our case, a sample is taken from the set of right and left offset vectors, **g_i,r_** and **g_i,l,_** corresponding to a single point, **x_i_**, taken at random. To calculate the certainty for any point **x_j_**, we find the neighbor closest to one of its four expected neighbor-locations given an exact fit to the model, **x_j_** ± **g_i,r_** and **x_j_** ± **g_i,l_**. The uncertainty is the distance between this neighbor and its expected location. The final model, consisting of **g_r_** and **g_l_,** is taken as the mean offset vector pair over the consensus set. Now, if we impose an exactly regular pattern on the sample, we would find the unit located at the *n*th column and *m*th row at an offset of *m* **g_r_**+*n* **g_l_** from the origin, where *m* and *n* are integers.

We select a detected location **x_i_** to assign to row 0 and column 0. Any location is as good as any other, so we may arbitrarily select **x_0_**. This location’s neighbors are traversed and assigned to the appropriate column and row, and in turn the neighbors of those locations are traversed, and so on until each location has a row and column associated with it that is consistent with all of its neighbors. For example, the neighbor located at **x_0_**+ **g_r_** would be assigned to column 1, row 0, and **x_0_** - **g_l_**, if it exists, would be at column 0, row −1. Let the *c_r,i_* and *c_l,i_* represent the column and row coordinates for location **x_i_** respectively, then the expected “real” location for **x_i_** is **y_i_** = **x_0_**+ *c_r,I_* **g_r_**+*c_l,I_* **g_l_**. Now we have a set **{x_i_}** of measured locations and a set **{y_i_}** of expected locations. The overall distortion may be modeled as a function ***T***: **y_i_** → **x_i_**. Although it is not possible to know how accurate our idealized model is with respect to “shear” and “stretch,” we can ignore this affine distortion because standard registration techniques are invariant to it. Let **{y’_i_}** be the result of affine alignment of **{y_i_}** to **{x_i_}**, then we take ***T***: **y’_i_** → **x_i_** to be the nonlinear distortion model for the tSEM.

## Results

### tSEM Image Quality is Comparable to TEM Images

**Figure 3:**
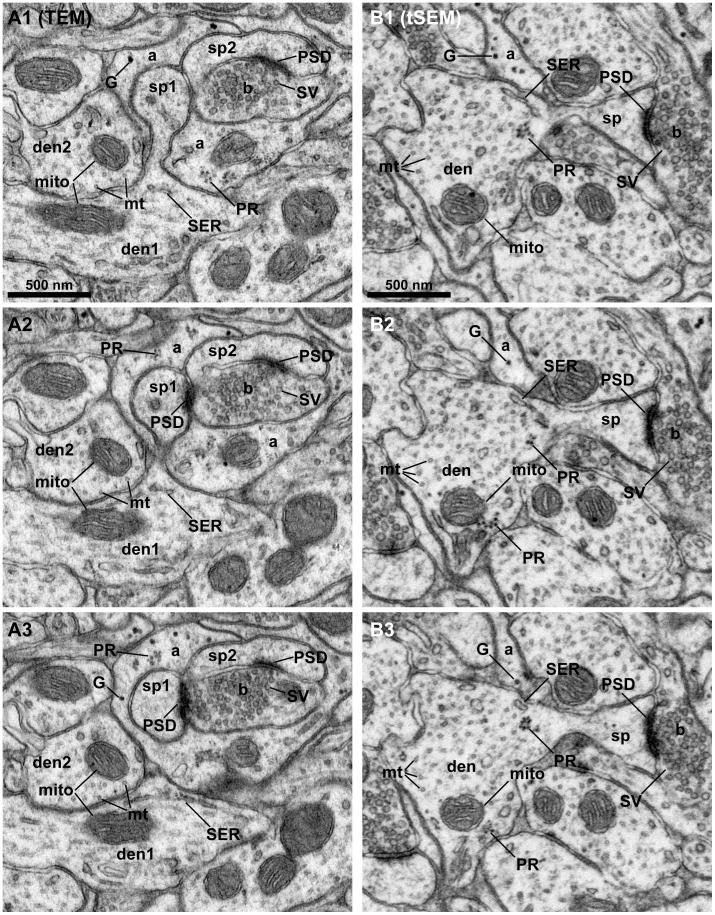
Quality comparison of images acquired on tSEM vs. TEM. Serial section images from the middle molecular layer of the hippocampal dentate gyrus acquired on a TEM (A1–3) and tSEM (B1–3). They were taken as 8-bit grayscale images, and the brightness and contrast were then adjusted to match the images from the two different EM platforms. A1–3: An obliquely cut dendrite (den1) gives rise to a mushroom-shaped spine (sp1) with postsynaptic density (PSD), making a synapse with an axonal bouton (b) containing synaptic vesicles (SV). This bouton also makes a synapse with another spine (sp2). These spines and bouton are wrapped around by an astrocytic process that contains glycogen granules (G) and polyribosomes (PR). A tubule of smooth endoplasmic reticulum (SER) and mitochondrion (mito) are located in the dendritic shaft. Cross-sectioned microtubules (mt) are clearly visible in an adjacent dendrite (den2), which also contains a mitochondrion (mito). B1–3: A mushroom-shaped spine (sp) on a dendrite (den) makes a synapse with a thickened PSD on an axonal bouton (b) containing synaptic vesicles (SV). A tubule of SER is visible at the base of this spine, along with a cluster of polyribosomes (PR). Cross-sectioned microtubules (mt) are also visible in this dendrite, which also contains a mitochondrion (mito). Clusters of polyribosomes (PR) are visible adjacent to a mitochondrion (in B2–3). Glycogen granules (G) are found in a neighboring astrocytic process (a). The original pixels are retained in all images in this figure. Only brightness and contrast were adjusted to match images acquired on the two EM platforms.

The new tSEM system accommodates specimens prepared in the same manner as for TEM. Compared to TEM, we observed qualitatively that the tSEM imaging was less prone to drift and shrinkage. The autofocus routine in tSEM repeatedly scans a small area in the center of the imaging field, which leads to greater brightening of the focus area compared to the rest of scan area (Fig. 1F). This brightening, however, does not cause loss of content; adjusting the brightness to the autofocus scan area and imaging in 16-bit mode retained the greyscale for later analysis of ultrastructure in this repeatedly scanned area (Fig. 1F2). Figure 3 demonstrates the tSEM image quality is comparable to TEM, and is excellent for identification, 3D reconstruction, and analysis of subcellular structures.

### Scan and Lens Distortions were Negligible in the tSEM System

Image distortions can affect calibration of pixel size and section thickness, which are critical steps in quantitative 3D analysis of tissue volumes. Pixel size was calibrated based on a grating replica image (Fig. 2A inset) that was imaged along with the serial section series. Section thickness is estimated with the cylindrical mitochondria method, which uses the ratio of the maximum diameter of longitudinally sectioned mitochondria (or other cylindrical objects) to the number of serial sections they span [51]. For our typical series acquired on either tSEM or TEM, the voxel size obtained through these methods is about 2 nm×2 nm×45 nm (x×y×z). This calibration is applied to the entire tissue volume for three-dimensional quantitative analysis of reconstructed neuropil and synapse structures (e.g., counting, lengths, area, volume, z-distances) that are sampled based on well-defined sets of structural criteria (see Discussion).

**Figure 4:**
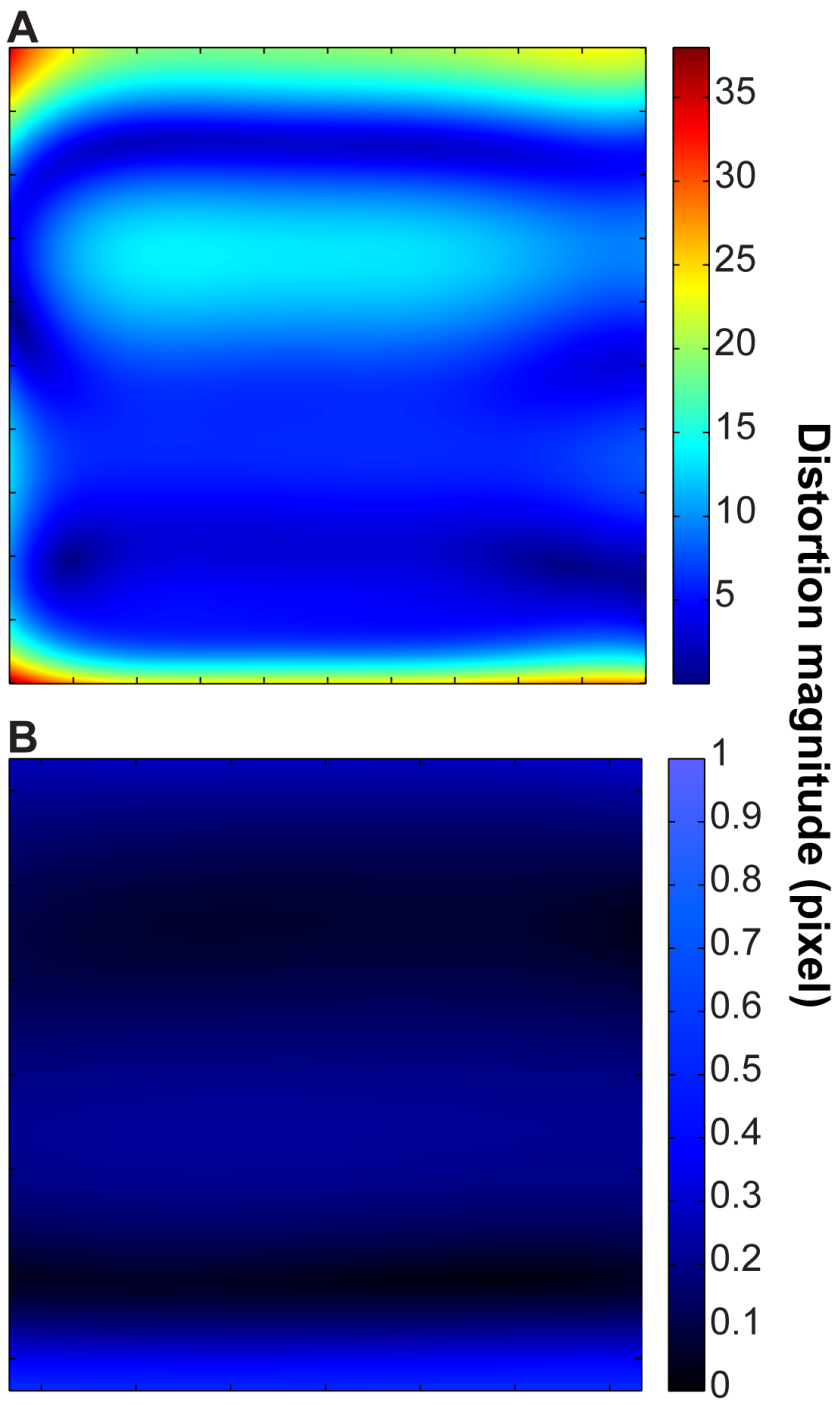
Image distortion analysis. A: tSEM full field distortion magnitude, corresponding to 24,000×24,000 pixel image. Maximum distortion magnitude is 37.93 pixel (rms = 9.68 pixel). This is equivalent to 0.04% rms distortion. B: tSEM field in A was cropped to the size equivalent TEM field (4,096×4,096 pixels). Note the scale bar is necessarily different. Maximum distortion magnitude is 0.55 pixel (rms = 0.19 pixel). This is effectively zero distortion, equivalent to 0.0047% rms distortion. Since we cannot accurately measure stretch and shear in the calibration replica, we ignore affine distortion modes.

Scan distortion was estimated using a bivariate polynomial model, created as described in the methods section. We calculated the transformed location for each pixel in the original image and measured the distance to its original location, after affine alignment. The root-mean-square (rms) of the distance measurements for the full 24,000×24,000 pixel field was found to be 9.68 pixels (Fig. 4A). This reduces drastically when the field is cropped to be similar to that obtained in the TEM. Over a 4,096×4,096 pixel field, we measured an rms distortion magnitude of only 0.19 pixels, which is negligible (Fig. 4B).

### Size of Single Image Fields in tSEM are much Greater than in TEM Cameras at the Same Pixel Size

**Figure 5:**
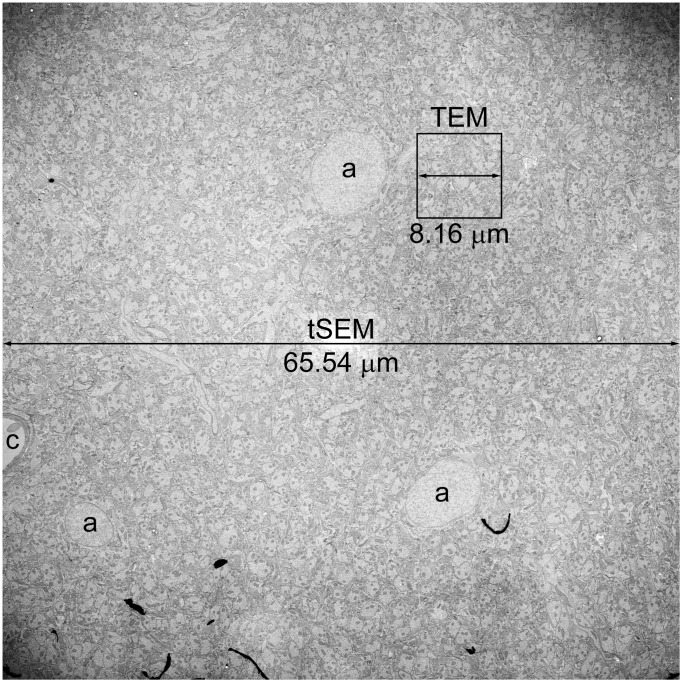
Field size comparison of images acquired on tSEM vs. TEM. This single field image of the rat hippocampal dentate gyrus (inner molecular layer) was acquired on tSEM originally at 32,768×32,768 pixels, or 65.54 µm×65.54 µm at 2 nm/pixel. Three astrocyte soma with round nuclei (a) and part of a capillary (c) are visible in this image. Boxed area indicates the size of a single field that can be imaged on our TEM (4,080×4,080 pixels, or 8.16 µm×8.16 µm at 2 nm/pixel). Note that the size of TEM field is similar to that of the nucleus of an astrocyte. The image has been adjusted for brightness and contrast, and re-sampled from the original pixel dimensions to 1,836×1,836 pixels during preparation of this figure.

The bottom mount CCD camera on our TEM obtains an image field of 4,080×4,080 pixels (i.e. ∼67 µm^2^ at 2 nm/pixel, obtained at 6,000 magnification), which is among the largest currently available. The conventional interface on a FE-SEM only obtains image fields of about 3,000×2,000 pixels (i.e. ∼28 µm^2^ at 2 nm/pixel); however, when integrated with the ATLAS system, the single tSEM image field can be at least 32,768×32,768 pixels (i.e. 4,295 µm^2^ at 2 nm/pixel). Thus, the tSEM readily images an area more than 60 times greater than the TEM (Fig. 5). The tSEM system with its large chamber and precise scan and stage control was used to automate acquisition of serial section images from these much larger field areas.

**Figure 6:**
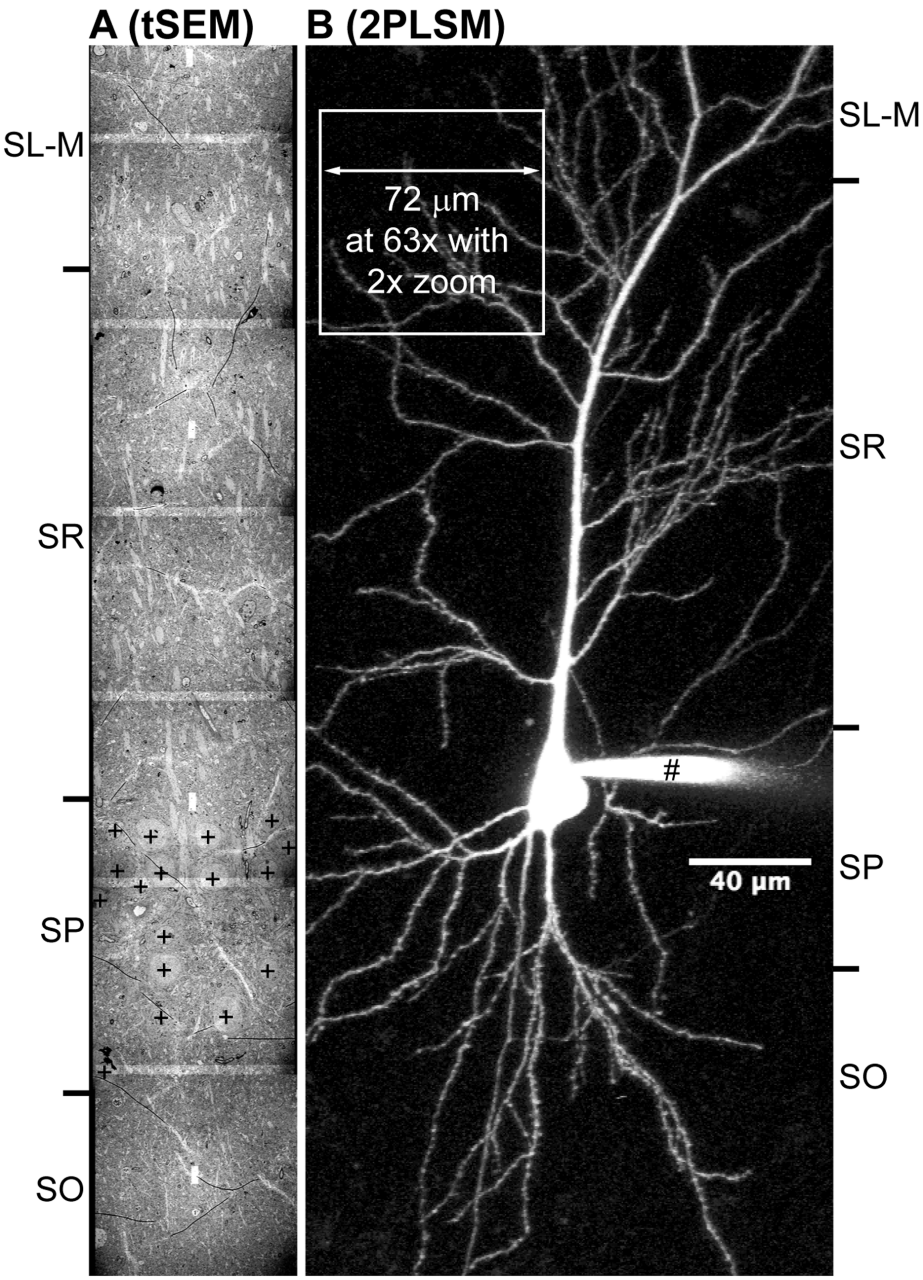
Field size comparison of images acquired on tSEM and two-photon laser-scanning microscope (2PLSM). A: A tSEM image containing a mosaic of 7 image tiles, from rat hippocampal area CA1, with the field size measuring 67 µm×399 µm. Overlaps between image tiles appear as lighter bands. Soma of the pyramidal neurons are indicated by “+”. The original image tiles were taken as 10×1 mosaic covering 608 µm×65 µm area (32,768×32,768 pixels per tile at 2 nm pixel size), encompassing all layers in the area CA1 (SO = Stratum Oriens; SP = Stratum Pyramidale; SR = Stratum Radiatum; SL-M = Stratum Lacunosum Moleculare). The image tiles were stitched together with Fiji/TrakEM2, down-sampled to 223 nm pixel size, rotated 90°, and cropped to 303×1792 pixels (67 µm×399 µm) to scale with the image in B. B: A pyramidal neuron in the rat (10-week old) hippocampal area CA1 was filled with Alexa 594 dye (40 µM) with a patch pipette (#). The original fluorescence image stack was acquired using laser tuned to 880 nm (Spectra Physics Mai Tai) on a 2PLSM (Leica SP 5 RS) with a 20× water immersion objective (N.A. = 1.0). Image field size was 1024×1024 pixels (455.88 µm×455.88 µm and 120 µm deep; 255 optical sections), which was then projected and cropped to 384×896 pixels (171 µm×399 µm). White box indicates calculated field for imaging with a 63× objective and 2× digital zoom (i.e., 455.88 µm/[63/20]/2 = 72.36 µm), which is on the order of single field size of a tSEM image. Scale bar is valid for both A and B. Image courtesy of R. Chitwood, Center for Learning and Memory, The University of Texas at Austin.

The imaging field size of the tSEM can be extended further by automated montaging through the ATLAS system (Figs. 1E and 6A). The montaging process in tSEM, by virtue of the larger field size, greatly reduces the total number of smaller images required and; hence, the total amount of edge-overlap than would be needed to produce a comparable montage area in TEM. It is possible now to take advantage of these automated acquisition and montaging capabilities in tSEM to photograph the entire face of much larger serial sections each ∼500 µm wide×>100 µm high (Figs. 1E and 6A), increased from ∼100 µm wide×30 µm high used in our previous studies [31]. The much larger tSEM field sizes can be used to automatically photograph and montage tissue volumes comparable to a typical confocal image volume (Fig. 6B), with much greater resolution to identify and measure synapses and other key subcellular structures (Fig. 3).

### Reduced Operator Involvement for Image Acquisition with tSEM Compared to TEM (Table 1)

In both TEM and tSEM, the total z-dimension of the imaged tissue volume is limited by the number of serial ultrathin sections obtained, which increases with operator skill. Collection of 200 serial sections is routine, and 1000 serial sections is possible for skilled operators, and about one day is required to prepare, cut and collect 200–1000 serial sections. For TEM, a single field image can be acquired rapidly (∼1 sec); however, there are many manual steps that multiply the operator time substantially and image acquisition across 200 serial sections requires several attended working **days**. First, the operator must insert one or a few specimen grids at a time in the TEM, and then find, align and stabilize the field of interest before acquiring each image. Alignment during image acquisition is critical for the small TEM field sizes because even a slight shift in positioning from section to section, caused by drift or operator error, can substantially reduce the region of interest collected on adjacent sections, and hence the tissue volume available for subsequent 3D analysis. Thus, the operator time for manual serial image acquisition in TEM is proportional to the total number of images required to generate the volume of interest. Some systems have been developed to automate the grid loading (e.g., [52] and Gatan Select 100™), but at present they still require a lot of operator attention. Even though automated TEM montaging (e.g., [15], [32]) has helped to reduce operator time, the process of montaging has its own issues including: (1) montaging many small field images produces substantial data redundancy and requires significant computing time; (2) electron dosage is not evenly distributed during montaging and hence can result in non-uniform distortion, requiring further post-image processing to correct the distortions, and (3) manual exchange of specimen grids can add substantial operator time for TEM montaging across serial sections. The new tSEM overcomes these cumbersome features of montaging in TEM.

In addition, the grid exchanges are minimized in the new tSEM system because the current specimen holder accommodates up to 12 grids at a time (easily 200 serial sections) and there is certainly room for a larger holder in the chamber. The attended operation involves simply locating, adjusting scan rotation, and marking the center of the imaging field on each serial section, which takes several hours (not days) for 200 serial sections. The rest of the image acquisition process is automated for image focus and brightness optimization, acquisition, and stage translation from one field to the next. Since the fields are so large, slight shifts in positioning of the center mark do not dramatically affect the tissue volume available for subsequent 3D analysis. Furthermore, operator time remains constant even when montaging to enlarge the field because only the center of the entire field need be marked per section to guide the automated acquisition (Fig. 1E).

### Image Artifacts are Balanced by Increased Image Area and Automated Elastic Alignment

Because tSEM uses serial ultrathin sections to achieve the desired axial resolution, the same limitations of TEM apply regarding handling of fragile serial section ribbons and grids [31]. A long ribbon of serial sections must be broken into shorter segments to fit them within the slot of a TEM grid. Sections must be supported on low-structure, electron transparent support film, such as Pioloform. Depending on the quality of the knife and skill of the operator, cutting forces can produce compression or knife marks and tears, settling of the sections on the film can produce folds, and sections can be lost, especially during ribbon breakup. These artifacts can interfere with accurate local alignment, making 3D reconstructions challenging or even impossible. Hence, in the past with a TEM, sections were first viewed through the entire series to find small regions where the fields could be imaged across serial sections while minimizing encounters with artifacts. The larger tSEM imaging field makes it harder to avoid these potential artifacts. The new elastic alignment tool in TrakEM2 [43] is much less sensitive to the section artifacts than prior alignment strategies, and provides accurate alignment across sections that would otherwise be distorted (Movie S1). Thus, the combination of automated image acquisition, afforded by the tSEM, and the enhanced elastic alignment tool, quickly provides much larger volumes for quantitative ultrastructural analysis.

### The tSEM System Images More, yet is Simpler, Smaller and More Affordable than the TEM

Table 1 further summarizes the main characteristics of TEM and tSEM. The tSEM is similar to TEM in the methods used to process the tissue and maintains the minimum nanoscale resolution needed to recognize and measure subcellular elements of the neuropil. The tSEM instrument is simpler and smaller compared to the TEM, at least partly because it does not require an image-forming lens below the specimen and the vacuum requirement for tSEM at 28 kV is less stringent than for the TEM operating at 120 kV. Thus, the tSEM occupies a smaller floor space that readily fits in a standard laboratory. Fully outfitted, the tSEM system price is about half the cost of a TEM with a large-format CCD camera, making tSEM more affordable for new and established investigators.

**Table 1:** Comparison of the new tSEM versus TEM systems.

		tSEM	TEM
	Platform	FE-SEM^a^	TEM
	Specimen holder	multiple grids at a time	usually one grid at a time (some special holders)
	Illumination	rastered probe	entire image field
	Accelerating voltage	28 kV	120 kV
	Detection method	transmitted electron detector	CCD camera
	Automation	*Routine*: Stage translation, image optimization (focus, brightness, etc.), image acquisition, and mosaic.	*Specialized*: Some modules for focus and astigmatism compensation, some mosaic functions.^b^
	Usual operator involvement	Identify center of image fields & set scan rotation on each section	Repeated specimen exchange & stage translation, physical specimen rotation, image optimization & acquisition
	Relative cost of instrument:	∼Half that of manual TEM	
**(a)**	Optimal pixel size (in x–y) for analysis	2 nm	2 nm
**(b)**	Section thickness	45 nm	45 nm
	Voxel size ( = **a**×**a**×**b**)	2 nm×2 nm×45 nm	2 nm×2 nm×45 nm
**(c)**	Typical number of serial sections	200	200
**(d)**	Maximum single field dimensions	32,768 pixels per side	4,080 pixels per side
**(e)**	Maximum single image field dimensions ( = **a**×**d**)	65.54 µm per side	8.160 µm per side
**(f)**	Maximum single image field area ( = **e**×**e**)	4,295 µm^2^	66.59 µm^2^
**(g)**	Volume of imaged tissue ( = **b**×**c**×**f**)	38,660 µm^3^	599.3 µm^3^
	Total number of voxels ( = **c**×**d**×**d**)	2.147×10^11^ voxels	3.329×10^9^ voxels
**(h)**	Operator time to acquire single field image over 200 sections	∼4 hr (plus additional ∼74 hr for unattended image acquisition).^c^	∼40 hr.
	Volume of imaged tissue per operator time ( = **g**/**h**)	9664 µm^3^/hr	14.98 µm^3^/hr

a At the time of this writing, Zeiss is the sole source of the FE-SEM system designed for high-resolution large-field transmission imaging of biological samples as described here.

b TEM can be retrofitted to acquire mosaic images automatically with open source software such as Leginon (National Resource for Automated Molecular Microscopy, The Scripps Research Institute; http://www.leginon.org/) and SerialEM (The Boulder Lab for 3D Electron Microscopy, University of Colorado; http://bio3d.colorado.edu/SerialEM/).

c Note that the operator time for tSEM remains constant for the same number of serial sections, even when the volume size is increased by montaging.

## Discussion

The new tSEM offers a cost-effective and labor-reducing approach to high-throughput ultrastructural imaging of biological specimens. It provides a high degree of automation that markedly reduces operator time during image acquisition while greatly increasing the size of the image fields and volumes without compromising image quality. The tissue volumes one can image with tSEM are comparable to those obtained through light microscopy, such as two-photon imaging, thereby providing a new and efficient platform to link live-imaging with ultrastructural analysis of underlying subcellular processes.

The tSEM system described here adds to a list of several new ssEM methods that have been developed recently to obtain dense volume reconstructions of brain neuropil in order to understand neural circuitry at the level of synaptic connections [15], [27], [28], [36]–[38,]. Each of these new ssEM approaches has advantages and disadvantages relative to ssTEM. Some of the advantages include larger image field size, finer axial resolution, reduced section loss, reduced physical and optical distortion, and automation of serial sectioning, imaging, alignment, and segmentation (for more comprehensive comparisons, see [36], [53]).

The use of non-destructive physical sectioning of the tissue for tSEM imaging has several advantages over the other SEM methods including: (1) The specimens can be archived for repeated tSEM imaging with larger field sizes or at higher resolutions. (2) The specimen can be used for high resolution post-embedding immunolabeling studies (fluorescent or gold) to localize molecules of interest in 3D (e.g., [22]). (3) The same specimen can be imaged with increased axial resolution by EM tomography to examine convoluted organelles buried within the thickness of a single ultrathin section, such as macromolecular complexes found, for example, in the presynaptic active zone (e.g., [54]). (4) Transmission imaging through ultrathin sections achieves greater lateral resolution compared to the backscatter imaging methods discussed below. (5) Sections are amenable to post-section staining for increased contrast. (6) Good ultramicrotomes are reasonably priced and commonly available.

The main disadvantage of the tSEM approach is that it still involves collection of ultrathin sections requiring substantial operator skill. Difficulties of serial ultrathin sectioning include: (1) Generating a long continuous ribbon of serial ultrathin sections with uniform thickness. (2) Dividing the ribbon into shorter segments that fit on the slot grids. (3) Avoiding breakage or folds in the fragile electron transparent substrates used to fill the slot and support the sections. (4) Reducing compression due to contact with the diamond knife. (5) Achieving uniform section thickness and avoiding contamination from local environment or poor preparation of post-section heavy metal stains. We have published numerous methods to avoid or minimize these potential flaws in serial ultrathin sections [31], [45]. Ultrathin sections supported on film materials such as polyimide and silicon nitride (e.g., [55]) that are more rigid and/or stable might reduce folds, and decrease further the fragility of the support. Such materials may also allow for larger specimen windows, reducing the need to break up ribbons into smaller segments.

Recently, several SEM-based serial section systems have been developed that avoid mounting ultrathin sections on slot grids. Two of these are serial imaging methods that are freed of the constraint of retaining intact sections, as well as facilitating a larger total axial imaging dimension. In these systems, backscattered electrons are detected to image directly from the block face that is serially removed either by a focused ion beam (FIB-SEM) [37], [38] or a diamond knife inside the SEM chamber (SBFSEM) [36]. Although FIB-SEM and SBSEM avoid ultrathin sectioning, the lateral resolution is currently lower than tSEM. Although microtubules and other small organelles have been detected in images acquired with FIB-SEM and SBFSEM [36], [37], our experience is that reliable identification and quantification, especially of microtubules, smooth-endoplasmic reticulum, and other small tubular structures in cross-section, becomes difficult or impossible at lower lateral resolutions [17], [19], [29], [30]. In addition, the image volumes obtained through FIB-SEM are currently too small to provide the circuit scale volumes achieved by the new tSEM system. Furthermore, the destructive nature of these block face imaging approaches does not allow for post-embedding immunolabeling, or for re-imaging of particular regions of interest at higher axial resolution through TEM tomography.

Low accelerating voltages are desirable for backscatter detection in that they improve lateral resolution, though at the expense of signal to noise ratio and dwell time. Another aspect of the backscatter imaging methods merits consideration: the depth of the tissue volume that interacts with the electron beam and emits the signal (i.e., backscattered electrons) depends on several parameters, including the accelerating voltage and the density of the tissue and stains. In the block face imaging methods, the signal depth does not always correspond with the thickness of the tissue removed by the knife or the FIB leaving open the possibility for oversampling from overlap of imaged tissue, or undersampling which would produce missing volumes where the amount of tissue removed is greater than that imaged on the block face. This potential for oversampling or undersampling of the tissue can affect quantitative analysis of small structures. In contrast, the projected images from the tSEM and TEM systems contain all of the objects, although some may be buried within the section depth. Since the sectioning and imaging in tSEM and TEM are non-destructive, re-examining small objects within the thickness of the sections can be done on the same grids using TEM tomography. In the future, it should be possible to exploit the robust control of stage, beam, and scan, as well as detector variety of tSEM to perform tomography [56] and improve axial resolution in the tSEM while performing large field imaging.

In other SEM-based backscatter imaging methods, serial ultrathin sections are collected on electron-opaque substrates, such as plastic tape (automatic tape collecting ultramicrotome or ATUM) [57], carbon-coated glass coverslips (array tomography) [28], or silicon wafers (serial section scanning electron microscopy or S^3^EM) [27]. In these applications, the issue of oversampling is mitigated by physical ultrathin sectioning of the tissue. However, lateral resolution may not be sufficient for the nanoscale analyses required for our studies. Specimen charging is known to affect lateral resolution in these low-voltage backscatter imaging methods, which may require additional carbon coating of the specimen (e.g., [28]). Charging can be reduced by the use of conductive silicon wafers [27] or carbon-coated glass coverslips [28] as specimen substrate. A recently developed method for *en bloc* heavy metal staining also helps to reduce specimen charging by making the biological specimen more conductive for block face imaging with backscattered electrons [58]. As noted above (Methods), we have not experienced charging of ultrathin sections prepared in the same manner as for TEM when imaged with tSEM at relatively high voltage (28 kV).

With the tSEM system, we were able to increase substantially the size of single-frame images by montaging with minimal total overlap. Another approach to increase the size of the imaging field has used a custom-built TEM with an array of four CCD cameras (TEMCA) to acquire image mosaics rapidly across individual serial ultrathin sections [15]. However, TEMCA requires substantial modification to the instrument (e.g., custom-built electron optics and camera systems) and specialized computational tools for image mosaicking and registration. Thus, the cost and limited availability of such a specialized custom-built system is prohibitive for most EM laboratories.

## Concluding Remarks

This new tSEM system is based on the detection of transmitted electrons on the FE-SEM platform and offers a cost-effective and labor-reducing method to obtain tissue volumes on the order of 10^4^–10^5^ µm^3^. This volume range is suitable for reconstruction of local circuits, such as determining synaptic relationships between an interneuron and neighboring pyramidal cells, or the composition of synapses in the neuropil within the domain of a single astrocyte. This level of analysis provides the opportunity to investigate many aspects of synaptic development and plasticity, while maintaining sufficient resolution to investigate changes in local subcellular components indicative of modified functions. Obviously, this new tSEM approach is not limited to investigation of brain tissue, but is generally applicable to all biological specimens prepared for standard ultrastructural analyses. Further improvements in computational tools will greatly facilitate 3D reconstruction and investigation of these large volumes.

## Supporting Information

Movie S1 **Serial tSEM images aligned with elastic alignment tool.** Here 201 serial section images were acquired from the rat dentate gyrus on tSEM at 16,384×16,384 pixels (2 nm/pixel), and aligned as described in Methods using Fiji/TrakEM2 software. The aligned images were then cropped to 12,830×6,861 pixels (25.66 µm×13.72 µm) and down-sampled to 1,920×799 pixels in Fiji/TrakEM2. The image histograms were adjusted using Fiji with the “Enhance Local Contrast (CLAHE)” plugin (http://fiji.sc/wiki/index.php/Enhance_Local_Contrast_%28CLAHE%29) before this movie (7 frames/sec) was generated. This movie demonstrates the effectiveness of the elastic alignment tool, where the presence of various section artifacts (e.g., folds and staining artifacts) does not affect the alignment of artifact-free regions across large serial tSEM images.
(ZIP)
